# Extracellular water/total body water ratio as predictor of mortality in hemodialysis patients

**DOI:** 10.1080/0886022X.2021.1922442

**Published:** 2021-05-10

**Authors:** Rosa Pérez-Morales, Javier Donate-Correa, Ernesto Martín-Núñez, Nayra Pérez-Delgado, Carla Ferri, Aurora López-Montes, Alejandro Jiménez-Sosa, Juan Francisco Navarro-González

**Affiliations:** a Nephrology Department, Hospital Universitario Nuestra Señora de Candelaria (HUNSC), Santa Cruz de Tenerife, Spain; b Research Unit, HUNSC, Santa Cruz de Tenerife, Spain; c Clinical Analysis Service, HUNSC, Santa Cruz de Tenerife, Spain; d Nephrology Department, Complejo Hospitalario Universitario de Albacete, Albacete, Spain; e Research Unit, Hospital Universitario de Canarias, La Laguna, Spain

**Keywords:** Bioimpedance, ECW/TBW, overhydration, hemodialysis, mortality

## Abstract

**Background:**

Overhydration is a predictor of mortality in hemodialysis (HD) patients. Bioimpedance spectroscopy (BIS) is used to determine the body composition. Extracellular Water/Total Body Water (ECW/TBW) ratio has been proposed to predict mortality.

**Methods:**

Multicenter, prospective, observational, proof-of-concept study to estimate the impact of ECW/TBW in global and cardiovascular mortality and the relationship with cardiovascular biomarkers. The study included 60 patients (mean age, 71.8 ± 11.4 years; mean time on HD, 52.3 ± 30.8 months) with a median follow-up of 30.5 months (IQ range, 17.2–34 months).

**Results:**

Post-dialysis ECW/TBW was directly associated with NT-proBNP and cTnT. During the study 28 patients died, most of them (43%) due to cardiovascular events. Compared to the survivors, these subjects had a higher post-dialysis ECW/TBW ratio (*p* = 0.006), while for cardiovascular mortality the only significant difference was a higher pre-dialysis ECW/TBW. The ability of post-dialysis ECW/TBW ratio to predict all-cause mortality had an area under the ROC curve (AUC) of 0.71 (CI 95%, 0.57–0.81; *p* = 0.002), with a cutoff point of 0.5023. For cardiovascular mortality the AUC was 0.66 (CI 95%, 0.52–0.77; *p* = 0.045), with a cutoff point of 0.4713.

**Conclusions:**

The post-dialysis ECW/TBW ratio measured by BIS can be a predictor of all-cause and cardiovascular mortality.

## Background

Bioelectrical impedance analysis (BIA) is a method for determining body composition [1]. Spectroscopic bioimpedance (BIS), has been validated against gold standard methods [2,3]. It is a promising tool for determining total body water in patients with pathological hydration [2–5], for distinguishing intracellular water (ICW) and extracellular water (ECW), and for estimating body composition in hemodialysis (HD) patients [2,5,6]. Overhydration is an independent predictor of mortality in chronic HD patients [7,8], while body water distribution has been associated with blood levels of diverse biomarkers, such as troponin (cTnT), N-terminal pro-brain natriuretic peptide (NT-proBNP) and C-reactive protein (CRP) [9,10]. High cTnT concentrations in asymptomatic patients have been associated with cardiovascular (CV) risk factors, left ventricular hypertrophy and coronary artery disease [11,12], and are considered an independent predictor of all-cause and cardiovascular mortality in chronic kidney disease (CKD) patients [11–14]. However, reduction in renal clearance and removal by HD adversely affects the level and utility of high-sensitivity troponin I for diagnosis of acute myocardial infarction, and about 25% of HD patients have an increment of troponin levels after dialysis, maybe due to myocardial injury related with high flow dialysis and high hemoglobin levels [15]. Park et al. [16] studied hypervolemia in HD patients, and used BIS to determine post-hemodialysis ECW/TBW (Extracellular Water/Total Body Water) ratio. They demonstrated the independent relationship between ECW/TBW and cTnT levels, a biochemical surrogate of cardiovascular mortality. On the other hand, NT-proBNP levels, which reflect the degree of volume overload and serve as an independent predictor of mortality in CKD patients [12,13,17], have been positively correlated with hypervolemia measured by bioimpedance in HD patients [9].

Bioimpedance–defined overhydration predicts mortality in dialysis patients regardless of the influence of comorbidity [8]. The classical definition for overhydration is an ECW greater than 15% [4,7]. The ECW/TBW has been suggested as an index of volume status in hemodialysis patients because excess volume accumulates mainly in ECW [18]. It is an index easy to use, intuitive, and well validated predictor of survival when comparing different approaches to using BIA-derived data to determine the hydration status in dialysis patients [19]. Nongnuch et al. [20] considered that HD patients were overhydrated when this ratio was ≥0.415, which was two standard deviations from the post-dialysis values. Volume overload defined as the ECW/TBW ≥0.40 may help to predict all causes of death in chronic hemodialysis patients [21]. Based on the knowledge gap regarding this issue, the aim of this proof-of-concept research was to study the relationship between ECW/TBW ratio, and the blood levels of cardiac and inflammatory biomarkers, as well as cardiovascular events and all-cause cardiovascular mortality.

## Methods

This was a multicentre, prospective, observational, proof-of-concept study conducted in three HD centers and coordinated by a tertiary care university hospital in Santa Cruz de Tenerife, Spain. The Institutional Review Board and Ethics Committee approved the study’s protocol (Reference number: 2011_27). All participants provided written informed consent before the study entry. Patients were considered eligible if they had been undergoing chronic HD for at least one year and were aged 18 years or over, and if they were clinically stable three months prior to inclusion. Exclusion criteria were infectious diseases (including positive tests for hepatitis B, C or HIV), active malignant or immunological diseases, cardiovascular or cerebrovascular events suffered in the month prior to inclusion; patients carrying pacemakers or coronary stents; subjects with kidney grafts; those with immunosuppressive treatment or immunomodulatory drugs, subjects with major amputations; and those with a body mass index (BMI) lower than 18 or higher than 40.

Patients underwent blood tests according to standard routines and procedures. Samples were stored at −80 °C for measurements of cTnT and NT-proBNP as cardiac biomarkers, and interleukin-6 (IL-6) as inflammatory biomarker. Both cTnT and NT-proBNP were determined by electrochemiluminescence immunoassay, with a range of reference of 0–5 pg/mL. IL-6 levels were measured by high-sensitivity ELISA (Human IL-6 Quantikine HS ELISA Kit, R&D Systems, Minneapolis, USA) using a 4-plate ELISA DSXTM processor (Vitro SA, Spain). The lowest detectable concentration level was 0.70 pg/mL. The accuracy of IL-6 readings was estimated by taking the mean coefficient of variation (CV) of three assays of 20-samples, with intra-assay and inter-assay CVs of 2.6 and 4.5%, respectively. The bioimpedance was performed with a portable BCM bioimpedance device (Fresenius Medical Care, Germany). The Fluid Management Tool software v.3.1.11 was used for calculating the different parameters. Measurements were made once, according to the manufacturer's instructions, before and after the midweek HD session conducted. The body composition variables and hydration biomarkers collected by BIS for the research were:

OH (L): Overhydration
ECW (L): Extracellular Water
ICW (L): Intracellular Water
TBW (L): Total Body Water
ECW/TBW: Ratio of Extracellular Water to Total Body Water
LTI (kg/m2): Lean Tissue Index
RFO (%): Relative Fluid Overload. Ratio of OH(L) to ECW(L)

Overhydration can be defined, according to classical criteria, either as an absolute value (in liters), or as a relative variable reflecting the excess of extracellular water and calculated by the following formula: Overhydration Percentage = OH(L)/ECW × 100%. Dialysis patients are considered overhydrated if the percentage of extracellular water is ≥15% [4,7]. Patients enrolled in the study were followed up prospectively. The information collected included demographic data, clinical and laboratory variables, kidney transplantations, referrals to other care centers, cardiovascular events requiring and not requiring hospital admission, as well as deaths due to cardiovascular and non-cardiovascular causes.

### Statistical analysis

Qualitative variables in the statistical analysis are expressed as frequencies and percentages. Quantitative variables are expressed as mean ± standard deviation or as medians and interquartile range (IR), as appropriate. Proportional comparisons were made with the Chi-squared and Fisher’s exact tests. Normality of variables was confirmed with the Kolmogorov-Smirnoff test. Inter-group comparisons were analyzed using the Mann–Whitney *U* or the Student’s *t* tests, as appropriate. Partial binomial logistic regression was used to estimate risk of mortality according to body composition ratios, controlling for the confounding variables that multivariate analysis had shown to be significant. Estimation of risk is expressed in odds ratios with a 95% confidence interval (CI). ROC curve analysis and Youden’s J statistic were used to estimate the optimal cut-off point for the post-dialysis ECW/TBW ratio. Finally, Kaplan-Meier analysis was used to estimate survival over time. Statistical analyses were performed using the SPSS v. 23.0 software package (IBM Corp., Armonk, NY, USA). Logistical regression analyses were performed with logXact v. 4.1 (Cytel Co., Cambridge, MA, USA). *p* Values below 0.05 were considered statistically significant.

## Results

From November 2011 to January 2013, 170 subjects were initially considered (Table 1). Finally, 60 Patients (73% men) met the inclusion criteria and accepted to participate in the study and were followed up prospectively until September 2015. The median observation time was 30.5 months (IR, 17.2–34 months). Patient characteristics are shown in Table 4. The BIS examination found pre-dialysis overhydration in 21 patients (35%), while this condition was maintained in 9 subjects (15%) after dialysis. Regarding hydration ratios, ECW/TBW and ECW/ICW were directly and significantly associated with NT-proBNP, both pre- and post-dialysis. A direct relationship with cTnT was also found, although only after dialysis. These ratios did not show any significant relationship with IL-6 levels (Table 2). The levels of these biomarkers for the cohort according to the cutoff point for global or cardiovascular mortality are shown in Table 3. Twenty-eight patients died during the study, the most common cause (43%) being cardiovascular events. Most of these subjects were males (71.4%) and had diabetes mellitus (57.1%). The age-adjusted Charlson index was 8.3 ± 1.8, and diabetic nephropathy was the most common etiology of kidney disease (42.9%). Deceased patients were older, had a higher Charlson Index, and were more likely to have a previous history of coronary disease, congestive heart failure (CHF) or peripheral vascular disease (PVD). On the other hand, they had a lower BMI and diastolic blood pressure (DBP). Iron therapy was significantly more common in patients who died (82.1 vs. 46.9%, *p* < 0.01). No differences in dialysis parameters were found between deceased and surviving patients (Table 4). Concerning bioimpedance parameters, deceased patients had lower TBW and ICW values, but higher pre-and post-dialysis ECW/TBW (Table 5). Regarding cardiovascular mortality, the only significant difference was found in pre-dialysis ECW/TBW, which was significantly higher in deceased patients (0.52 ± 0.05 vs.0.50 ± 0.04, *p* = 0.04). Finally, no difference was observed when comparison was performed according to smoking habit (smokers, 0.486 ± 0.0405 vs nonsmokers, 0.498 ± 0.0309, *p* = 0.36).

**Table 1.** Comparison of excluded/included clinical and demographic characteristics of the patients.

	Cases *n* = 60	Excluded *n* = 110	*p*-Value
Age (years)	72 ± 11	67 ± 15	0.04
Sex (male) – no. (%)	44 (73.3)	70 (63)	0.20
Height (cm)	167 ± 9	166 ± 9	0.43
Body mass index	26.87 ± 4.48	26.9 ± 4.49	0.92
Hypertension – no. (%)	50 (84.7)	81 (87.1)	0.68
Diabetes mellitus – no. (%)	33(55)	56 (60.2)	0.75
Smoking status – no. (%)	12 (20)	15 (16.1)	0.86

**Table 2.** Correlation between NT-proBNP and hydration ratios pre and post-dialysis.

	ECW/TBW pre-dialysis	ECW/TBW post-dialysis	E/I pre-dialysis	E/1 post-dialysis
NT-proBNP				
Rho	0.32	0.48	0.32	0.48
*p*	0.01	<0.001	0.01	<0.001
cTnT				
Rho	0.21	0.45	0.21	0.45
*p*	0.12	<0.001	0.11	<0.001
IL-6				
Rho	0.01	0.13	0.02	0.13
*p*	0.96	0.33	0.89	0.33

TBW: total body water; ECW: extracellular water; ICW: intracellular water; NT-proBNP: N-terminal pro-brain natriuretic peptide; cTnT: Troponin T; IL-6: Interleukin 6.

**Table 3.** Biomarker levels and ECW/TBW ratio cutoff for global and cardiovascular mortality.

Cardiovascular mortality			
ECW/TBW ratio cutoff	<0.4713	≥0.4713	*p*
NT-proBNP	1588 (704–4177)	5324 (2795.3 − 14,566)	0.001
cTnT	34.6 (24 − 49.4)	67.9 (48.3–91.9)	<0.001
IL-6	5.7(3.2 − 9.9)	6.5 (2.9 − 10.9)	0.54
Global mortality			
ECW/TBW ratio cutoff	<0.5023	≥0.5023	*p*
NT-proBNP	3084 (1529–6392.8)	7076.5 (3436.5–17,808)	0.02
cTnT	53.4 (35.2–77.2)	67.9 (51.6–92.7)	0.05
IL-6	6.1 (2.8–10.2)	3.1 (6.4–11.5)	0.77

Results are expressed with median (P_25_–P_75_).

**Table 4.** Demographic and clinical characteristics of the 60 patients included in the study, survivors and non survivors.

	Cohort *n* = 60	Survivors *n* = 32	Non survivors *n* = 28	*p*
Sex (men) – n (%)	44 (73.3)	24 (75)	20 (71.4)	0.76
Age (years)	71.8 ± 11.3	67.9 ± 11.2	76.3 ± 9.9	0.004
Cardiovascular background-n (%)				
Hypertension	50 (83.3)	29 (90.6)	21 (75)	0.17
Diabetes mellitus	33 (55)	17 (53.1)	16 (57.1)	0.75
Previous cardiovascular events	41 (68.3)	19 (59.4)	22 (78.6)	0.11
Arrhythmia	5 (8.3)	2 (6.3)	3 (10.7)	0.66
Coronary heart disease	11 (18.3)	2 (6.3)	9 (32.1)	0.010
Congestive heart failure	11(18.3)	1 (3.1)	10 (35.7)	0.001
Cerebrovascular disease	16(26.7)	9 (28.1)	7 (25)	0.78
Peripheral vascular disease	20 (33.3)	7 (21.9)	13 (46.4)	0.003
Toxic habits – n (%)				
Smoking:				
Never	32(53.3)	21 (65.6)	11 (39.3)	0.95
Former smoker	16(26.7)	6 (18.8)	10 (35.7)	
Smoker	12(20)	5 (15.6)	7 (25.5)	
Alcoholism:				
Never	43(71.7)	21(65.6)	22 (78.6)	0.47
Former drinker	7(11.7)	4 (12.5)	3 (42.9)	
Active drinker	10(16.7)	7(21.9)	3 (10.7)	
Etiology of renal disease – n (%)				
Diabetic nephropathy	25(41.7)	13 (40.6)	12 (42.9)	0.81
Unknown	12 (20)	6 (18.8)	6 (21.4)	
Nephroangiosclerosis	7 (11.7)	5 (15.6)	2 (7.1)	
Polycystic renal disease	6 (10)	3 (9.4)	3 (10.7)	
Ischemic nephropathy	6 (10)	2 (6.3)	4 (14.3)	
Tubule-interstitial nephropathy	2 (3.3)	1 (3.1)	1 (3.6)	
IgA glomerulonephritis	1(1.7)	1 (3.1)	0 (0)	
IgG glomerulonephritis	1(1.7)	1 (3.1)	0 (0)	
Blood pressure (mmHg)				
Systolic	137.72 ± 23.89	139.7 ± 20.9	135.46 ± 27.05	0.50
Diastolic	73.28 ± 16.22	77.7 ± 16.05	68.25 ± 15.16	0.023
Pharmacological treatment – n (%)				
Antihypertensive	39 (65)	20 (62.5)	19 (67.9)	0.66
Lipid-lowering	39 (65)	21 (65.6)	18 (64.3)	0.91
Erithropoiesis stimulating agents	51 (85)	25 (80.6)	26 (92.9)	0.162
Iron therapy	38 (63.3)	15 (46.9)	23 (82.1)	0.005
Dialysis vintage	52.33 ± 30.83	51.06 ± 30.3	53.79 ± 31.9	0.694
Dialysis session time – *n* (%)				
Less than 12 h	2 (3.3)	0 (0)	2 (7.1)	0.232
12 h	48 (80)	25 (78.1)	23 (82.1)	
More than 12 h	10 (16.7)	7 (21.9)	3 (10.7)	
Kt /V – *n* (%)				
Under 1.3	11(18.3)	5 (15.6)	6 (21.4)	0.562
Greater than 1.3	49(81.7)	27 (84.4)	22 (78.6)	
EPO resistance index – *n* (%)	10(16.7)	3 (9.4)	7 (25)	0.046
Vascular access - *n* (%)				
Cuffed catheter	8(13.3)	5 (15.6)	3 (10.7)	0.42
Autologous arteriovenous fistula	50(83.3)	26 (81.3)	24 (85.7)	
Prosthetic arteriovenous fistula	2(3.3)	1 (3.1)	1 (3.6)	
Type of hemodialysis – *n* (%)				
Standard	59(98.3)	31 (96.9)	28 (47.6)	0.34
On-line	1(1.7)	1 (3.1)	0 (0)	
RFO	0.10 ± 0.114	0.087 ± 0.112	0.12 ± 0.115	0.15

RFO: relative fluid overload.

**Table 5.** Relationship between hydration parameters measured by bioimpedance in survivors and non survivors.

	Survivors (*n* = 32)	Non Survivors (*n* = 28)	*p*
TBW [L] pre-dialysis	36 ± 6.7	33 ± 5.2	0.02
ECW [L] pre-dialysis	18 ± 3.6	17 ± 2.3	0.13
ICW [L] pre-dialysis	18 ± 3.7	16 ± 3.5	0.006
TBW [L] post-dialysis	34 ± 6.0	30 ± 4.5	0.01
ECW [L] post-dialysis	16 ± 3.0	15 ± 2.2	0.14
ICW [L] post-dialysis	18 ± 3.5	15 ± 2.8	0.003
ECW/TBW pre-dialysis	0.492 ± 0.039	0.499 ± 0.049	0.07
ECW/TBW post-dialysis	0.479 ± 0.0366	0.508 ± 0.035	0.006

TBW: total body water; ECW: extracellular water; ICW: intracellular water; SD: standard deviation.

No difference was found in pre-dialysis Relative Fluid Overload between surviving (0.087 ± 0.112) and non-surviving patients (0.12 ± 0.115); *p* = 0.15 (Table 4). There were also no differences observed in the nutritional parameters taken by BIS in terms of survival, although pre- and post-dialysis. LTI values were lower in deceased patients. No relationship was found between low LTI and all-cause or cardiovascular mortality.

### ECW/TBW ratio and mortality

**Figure 1.**
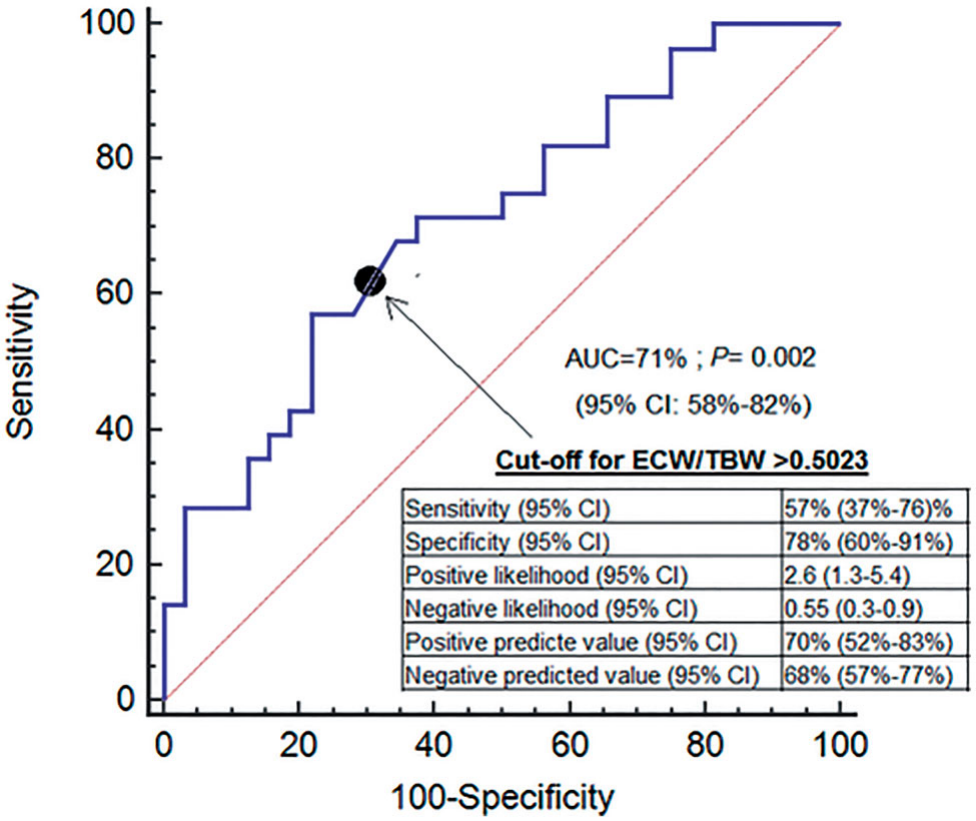
ROC curve for ECW/TBW using death as event.

**Figure 2.**
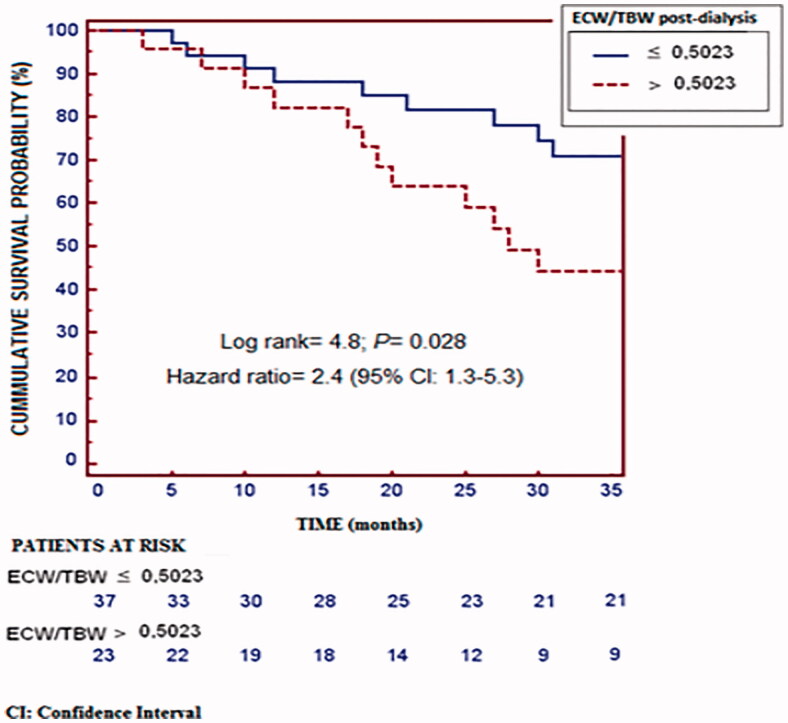
Kaplan–Meier survival curves using the cutoff ECW/TBW >0.5023. as predictor and death as outcome.

**Figure 3.**
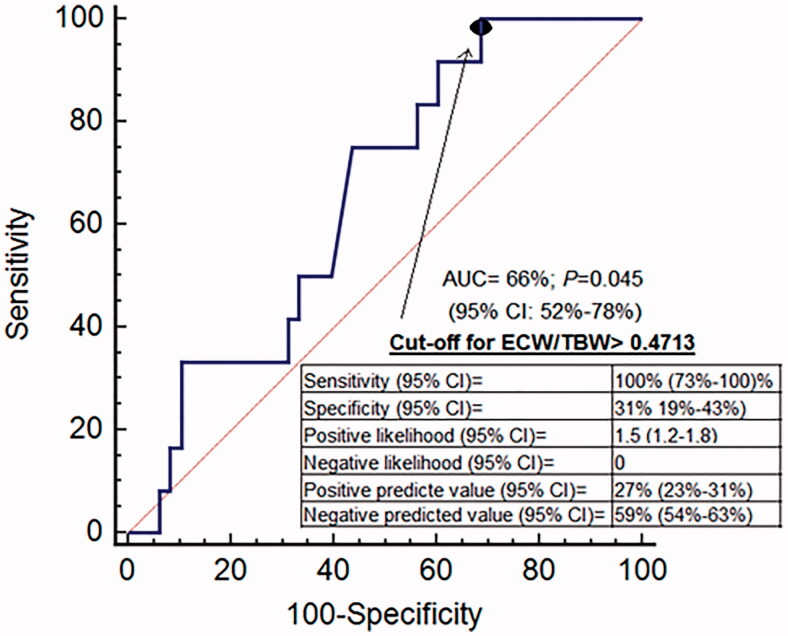
ROC curve for ECW/TBW using cardiovascular death as event.

**Figure 4.**
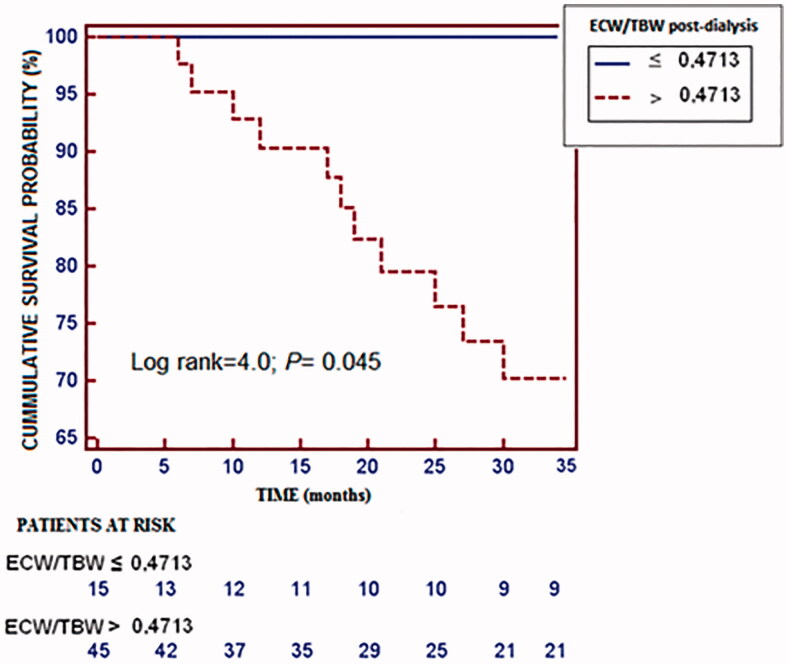
Kaplan–Meier survival curves using the cutoff ECW/TBW >0.4713 as predictor and death as outcome.

The predictive ability of post-dialysis ECW/TBW for all-cause mortality returned an AUC of 0.71 (95% CI, 0.57 to 0.81; *p* = 0.002). The value with the highest sensitivity (57%), specificity (78%) and Youden’s J score for predicting the likelihood of death was 0.5023 (Figure 1). Kaplan–Meier analysis revealed that patients with post-dialysis ECW/TBW lower than 0.5023 had a higher probability of survival (Figure 2). Patients with post-dialysis ECW/TBW ≥0.5023 were older, scored higher on the Charlson index, and were more likely to have type 2 diabetes mellitus (DM2) and a history of CHF. The optimal cutoff percentile for the ECW/TBW ratio (0.5023) corresponds to the 65th percentile. The ability of post-dialysis ECW/TBW for predicting cardiovascular mortality had an AUC of 0.66 (95% CI, 0.52 to 0.77; *p* = 0.045). The value with the highest sensitivity (100%), specificity (31%) and Youden’s J score for predicting the probability of death was 0.4713 (Figure 3). The likelihood of survival was higher with post-dialysis ECW/TBW ratio below 0.4713 (Figure 4). Patients with a ratio ≥0.4713 were older and more likely to have been smokers and have a history of cardiovascular disease. Post-dialysis ECW/TBW did not demonstrate a predictive value for cardiovascular events [AUC 0.517 (CI 95% 0.380–0.644; *p* = 0.087)].

Twenty-three patients (38.3%) had a post-dialysis ECW/TBW ratio higher than 0.5023, while 45 subjects (75%) had a post-dialysis ECW/TBW ratio higher than 0.4713. When analyzing total mortality, 16 patients (69.6%) with a post-dialysis ECW/TBW ratio higher than 0.5023 died, while among subjects with a ratio lower than 0.5023 there were 12 deaths (32.4%).

### Multivariate analysis

In order to obtain multiple regression models, the relationship between overhydration and mortality was studied after controlling for variables that had a *p* value <0.20 (age, BMI, Charlson Index, hypertension, diabetes, arrhythmias, coronary disease, congestive heart failure, cerebrovascular disease, peripheral vascular disease, DBP, iron therapy and EPO resistance). Congestive heart failure was excluded from the logistic regression analysis since it is part of the causal chain of death. Binomial logistic regression analysis showed a relationship between post-dialysis ECW/TBW and all-cause mortality. Cox regression analysis for all-cause mortality showed that the hazard ratio increased significantly when the post-dialysis ECW/TBW ratio value was 0.5023 or higher, but not remaining significant after adjusted for other variables (age, Charlson Index and time on dialysis) (Table 6). Regarding cardiovascular mortality, the hazard ratio was not statistically significant.

**Table 6.** Partial Cox regression models to predict global mortality using an ECW/TBW post-dialysis cutoff value higher than 0.5023 as the main independent variable.

	Hazard ratio	95% CI	*p*
Model 1			
ECW/TBW >0.5023	2.46	1.16–5.20	0.02
Model 2			
ECW/TBW >0.5023	2.05	0.94–4.48	0.07
Age (years)	1.03	0.99–1.08	0.15
Model 3			
ECW/TBW >0.5023	2.05	0.93–4.48	0.07
Charlson Index	1.19	0.96–1.46	0.11
Model 4			
ECW/TBW >0.5023	2.01	0.91–4.47	0.09
Charlson Index	1.18	0.94–1.47	0.15
Time in hemodialysis (months)	0.90	0.35–2.33	0.83

## Discussion

The main finding of our study is that post-dialysis ECW/TBW measured by BIS can be a useful parameter for predicting all-cause mortality in HD patients, with a 0.5023 cutoff point.

Bioimpedance has been used in clinical studies for decades, but only recently the impact of hyperhydration on survival has been demonstrated [7,8,22–24]. Wizemann et al. [7] found that baseline overhydration, in HD patients that an OH level above 2.5 L (OH:ECW ratio >15%) was associated with a significantly increased risk of mortality. Tabinor et al. [8] establishes in a meta-analysis that Bioimpedance-defined overhydration as a predictor of survival in end stage kidney failure (EEKF), regardless of the effect of malnutrition, inflammation, multimorbidity and cardiac structural disease.

Likewise, Chazot et al. [23] found that all-cause mortality in HD was significantly increased in the hyperhydrated patients while no difference in mortality was found among non-hyperhydrated. The ECW/TBW ratio has been established in several studies as an indirect measure of overhydration [22,25,26]. Kim et al. [21] found that a chronic fluid overload defined as a pre-dialysis ECW/TBW ≥0.40, could be a predictor for death from any cause, this cutoff value is based on a fluid status measurement in normal healthy Koreans (suggested by the manufacturer, Biospace, Seoul, South Korea). However, ECW/TBW of 0.40 is not an absolute cutoff for defining volume overload, because it can be affected by various factors including age, sex and comorbidities. This study suggests an individualized approach for application in clinical practice until further studies establish an optimal cutoff value for diagnosing volume overload in HD patients. Zoccali et al. [27] have demonstrated in a study with 39.566 hemodialysis patients that prolonged fluid overload was strongly associated with mortality. We did not found difference in pre-dialysis fluid overload between surviving and non-surviving patients; however, the main finding of our study shows that post-dialysis ECW/TBW measured by BIS can be a useful parameter for predicting all-cause mortality in HD patients, with a 0.5023 cutoff point. Recently, the Kidney Disease Outcomes Quality Initiative (KDOQI) Nutrition Guidelines in CKD had suggested using post-dialysis bioimpedance to assess body composition in adults with CKD 5D on Maintenance HD [28] Kim et al. compared BIA components between HD subjects and propensity score-matched controls, and investigated how BIA components changed before and after HD. They concluded that estimated normal hydrated weight using ECW/TBW can be a good marker for determining dry weight [29]. Post-dialysis ECW/TBW could better reflect prolonged overhydration than RFO, because ECW decrease after dialysis, modifying RFO (OH lt/ECW × 100) value more than ECW/TBW.

This ratio has been associated with cardiac biomarker levels related to mortality, such as NT-proBNP and cTnT [16,20,30]. Jacobs et al. [9] who conducted a prospective study in 44 HD patients who were followed-up for 6 months performed pre- and post-dialysis bioimpedance measurements and determined baseline levels of NT-proBNP, cTnT and C-reactive protein. The authors concluded that ECW/Body Weight ratio was directly related with NT-proBNP levels. In a study of 375 stable HD patients, Nongnuch et al. [20] found that pre-dialysis ECW/TBW ratio and relative overhydration were significantly higher in the uppermost NT-proBNP quartile compared to the lowest quartile. Similarly, to these previous studies, the results in our work show a direct and significant correlation between NT-proBNP and pre- and post-dialysis ECW/TBW. Concerning cTnT, Park et al. [16] studied the relationship between this biomarker and the BIS-estimated ECW/TBW ratio in 74 HD patients with no history of ischemic heart disease. They found a positive correlation between post-dialysis ECW/TBW and cTnT, which remained statistically significant after multivariate analysis. The population of our study presents evident differences in comparison with that from the study by Park et al. [16]; older subjects, higher number of male and diabetic patients, and longer HD. However, despite these differences, our findings also revealed a direct correlation between cTnT and post-dialysis ECW/TBW. This seems to confirm previous data regarding the usefulness of this ratio as a predictor of mortality. Moreover, even though our research findings were similar to those reported by Park et al. [16] and Nongnuch et al. [20] regarding the direct and significant relationship between post-dialysis ECW/TBW and NT-proBNP and cTnT levels, these biomarkers were not significantly related to survival. This may suggest that overhydration measured using the post-dialysis ECW/TBW ratio could be a better predictor of mortality than increased cardiac biomarkers, which is similar to the study of Tangvoraphonkchai and Davenport [31], who found that high ECW/TBW was associated with increased mortality in hemodialysis patients, although N-T-proBNP was not. In a prospective study of 753 HD and peritoneal dialysis patients who were followed up for 16 months, Paniagua et al.[24] demonstrated that the ECW/TBW ratio was a predictor of cardiovascular mortality regardless of the type of dialysis, whilst showing a marginal usefulness as a predictor of all-cause mortality. This last result may have been related to the heterogeneous nature of the sample in terms of age, BMI, viral liver infections, dialysis technique, and non-cardiovascular causes of death. Our research suggests the potential value of ECW/TBW as a predictor of all-cause mortality, has a more homogeneous patient sample in terms of dialysis technique, age and BMI, whilst excluding patients who tested positive for hepatitis B, C or HIV, and those with underlying inflammatory conditions to avoid confounding factors.

The classical criteria for defining relative overhydration and predicting mortality is the presence of an extracellular water higher than 15%.[7] Some authors suggest different cutoff points for this indicator, such as Onofriescu et al. [32] (>17.4%), while Vega et al. [33] proposed reducing the limit of overhydration measured by bioimpedance to 10%, since this value has been related to a reduction in the concentration of prealbumin and fat tissue in HD. Unlike relative overhydration, there is not currently a well-defined cutoff point of reference for overhydration measurement by the ECW/TBW ratio.

Nongnuch et al. [20] proposed a value ≥0.415, or two standard deviations from the post-dialysis values. Kim et al. [21] found a pre-HD cutoff point of ECW/TBW ≥0.40. Our research makes a contribution by proposing a similar cutoff point for considering this ratio as a predictor of all-cause and cardiovascular mortality. Thus, the post-dialysis ECW/TBW cutoff value for all-cause mortality was ≥0.5023, while for cardiovascular mortality the cutoff value was ≥0.4713.

Finally, post-HD bioimpedance measurements of overhydration appear to be more clinically relevant than pre-dialysis parameters, possibly because we had failed to correct fluid overload after dialytic treatment. Likewise, Tangvoraphonkchai and Davenport [31], suggest BIS should be performed preferably post-dialysis, when patients should be less overhydrated and have less electrolyte imbalance. MONDO study [34] included 8883 patients with a pre-dialysis multifrequency bioimpedance spectroscopy measurement in the year 2011, found estimated post-dialysis fluid depletion was associate with improved survival.

We acknowledge several limitations to this investigation. The small sample size, which might imply that some of our results did not reach statistical significance and limit the ability to describe all factors that influence the relationship between ECW/TBW and mortality. Since only 28 mortality events occurred, to avoid an overfitting effect we can only include 2–3 variables in each regression model. The explorative nature of the study determines that the conclusions cannot be considered definitive, while serving to generate hypothesis. The analysis was performed with one baseline and not with serial measurements, which could have been extremely relevant to better understand the relationship between overhydration and survival. Finally, since most subjects were older males, this can limit the extrapolation of our conclusions to patients with other characteristics. It is not clear that these analyses will definitely be more generalizable, but it at least will provide a more robust assessment of the association between the available data and mortality.

## Conclusions

The results of our study indicate that post-HD-bioimpedance measurements of overhydration in HD appear to be relevant. Furthermore, post-dialysis ECW/TBW obtained by BIS could be a good predictor of all-cause and cardiovascular mortality. Further long-term prospective studies on other cohorts are required to confirm the usefulness of the ECW/TBW cutoff values proposed in this study as predictors of mortality in HD patients.

## Data Availability

Materials described in the manuscript, including all relevant raw data, will be freely available to any scientist wishing to use them for noncommercial purposes, without breaching participant confidentiality.
